# Medical gas plasma-stimulated wound healing: Evidence and mechanisms

**DOI:** 10.1016/j.redox.2021.102116

**Published:** 2021-08-28

**Authors:** Sander Bekeschus, Thomas von Woedtke, Steffen Emmert, Anke Schmidt

**Affiliations:** a ZIK Plasmatis, Leibniz Institute for Plasma Science and Technology (INP), A Member of the Leibniz Research Alliance Leibniz Health Technology, Felix-Hausdorff-Str. 2, 17489, Greifswald, Germany; b Institute for Hygiene and Environmental Medicine, Greifswald University Medical Center, Sauerbruchstr., 17475, Greifswald, Germany; c Clinic for Dermatology and Venereology, Rostock University Medical Center, Strempelstr. 13, 18057, Rostock, Germany

**Keywords:** Defective healing, Dermatology, Plasma medicine, Reactive oxygen and nitrogen species, RNS, ROS, Ulcers

## Abstract

Defective wound healing poses a significant burden on patients and healthcare systems. In recent years, a novel reactive oxygen and nitrogen species (ROS/RNS) based therapy has received considerable attention among dermatologists for targeting chronic wounds. The multifaceted ROS/RNS are generated using gas plasma technology, a partially ionized gas operated at body temperature. This review integrates preclinical and clinical evidence into a set of working hypotheses mainly based on redox processes aiding in elucidating the mechanisms of action and optimizing gas plasmas for therapeutic purposes. These hypotheses include increased wound tissue oxygenation and vascularization, amplified apoptosis of senescent cells, redox signaling, and augmented microbial inactivation. Instead of a dominant role of a single effector, it is proposed that all mechanisms act in concert in gas plasma-stimulated healing, rationalizing the use of this technology in therapy-resistant wounds. Finally, addressable current challenges and future concepts are outlined, which may further promote the clinical utilization, efficacy, and safety of gas plasma technology in wound care in the future.

## Abbreviations

Ar: Argon
CAT: catalase
He: Helium
HMOX1, HO-1: Heme oxygenase 1
IL-10: Interleukin 10
IL-1β: Interleukin 1beta
IL-6: Interleukin 6
IL-8: Interleukin 8
KGF: Keratinocyte growth factor
MMPs: Matrix metalloproteinases
MPO: Myeloperoxidase
NO: Nitric oxide
NOS: Nitric oxide synthase
NOX: NADPH oxidase
NQO1: NAD(P)H dehydrogenase [quinone] 1
Nrf2: Nuclear factor erythroid 2-related factor 2
p53: Tumor suppressor protein p53
RNS: Reactive nitrogen species
ROS: Reactive oxygen species
SOD1: Sodium dismutase 1
TGFβ: Tumor necrosis factor beta
TIMPs: Inhibitors of matrix metalloproteinases
TNFα: Tumor necrosis factor-alpha

## 1 Introduction

Defective wound healing is probably the oldest medical condition in human history. In the pre-antibiotic age, even seemingly minor tissue damage combined with persistent infection had decided about life or death [1]. Wound management has, therefore, a long-standing tradition across the globe and cultures. With the onset of the antibiotic age, wound and infection-related deaths declined. Nowadays, most chronic wounds are no a consequence of battle and combat situations but are associated with aging and underlying diseases such as diabetes, vascular disease, pressure ulcers during extended hospitalization [2], and immunosuppression-associated infection [3]. In a meta-analysis, evidence-based benefits have been shown for some wound healing methods and the basic principles of local therapy for chronic wounds of venous leg ulcers [4]. However, therapeutic modalities being available and targeting defective wound healing are numerous, with a relatively low evidence level of many approaches [5,6]. Further complicating wound management, there is a widespread polypragmasia in the type and sequence of therapies [7]. Consequently, research on wound healing and management remains active, with new concepts and modalities continuously arising.

In the early 2000s, Chandan K. Sen set out the concept of redox control in wound healing [8]. The hypothesis was based on the findings that augmented generation of reactive oxygen species (ROS), especially superoxide (O_2_^−^) and hydrogen peroxide (H_2_O_2_), promoted the release of vascular endothelial growth factor (VEGF) and wound healing, resulting in improved hyper-proliferative epithelial regions, cell densities, deposition of connective tissue, and histological architecture [9]. Over the following years, the concept of redox signaling in wound healing [10] and the requirements of oxygen and oxygen-derived reactive species as an essential component of several wound healing-related processes was elaborated in greater detail [11]. Another conclusion from these studies is that controlled production and release of ROS and reactive nitrogen species (RNS) might promote wound healing. To this end, it is conceivable that ozone wound treatment is a longstanding concept for decades [12,13], albeit it has not entered widespread clinical practice. Other wound healing products such as neomycin/catalase (Pulvo 47) [14], dimethyl sulfoxide (DMSO) [15], and iron chelators [16] are intertwined with ROS production or removal as well. At about the same time, novel technology concepts emerged, capable of generating a plethora of ROS/RNS simultaneously in a localized and time-restricted manner and operated at body temperature: medical gas plasma-generating systems, intended for supporting the healing of wounds [17]. Since then, much progress has been made, and evidence and mechanisms of gas plasma-stimulated wound healing are reviewed in this work, which focuses on wounded skin rather than other fields in which plasma technology is envisioned to be employed for improving healing such as implantology, dentistry, and tumor wounds in oncology.

## 2 Wound healing and gas plasma technology

### 2.1 Wound healing

In life, tissue injury is inevitable. This is of crucial importance where microorganisms invade compromised tissues and cause infection. For this, tissues display high plasticity and the ability to seal a gap while bacteria are being removed by immune cells – to heal.

**Fig. 1:**
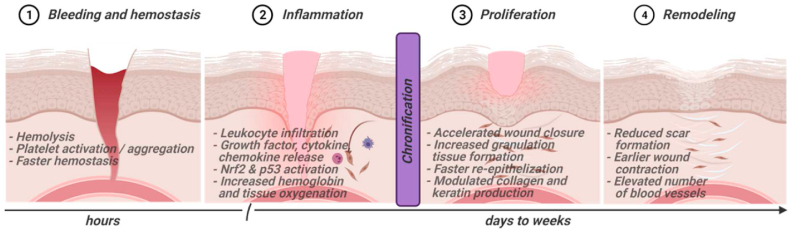
Sequence of wound healing stages and effects of gas plasma treatment during these processes. The length of the phases is not proportional. Many events were observed to be gas plasma treatment time-dependent. Created with BioRender.com.

The wound healing process is characterized by four continuous, overlapping, and precisely programmed phases: hemostasis, inflammation, proliferation, and remodeling [18]. Hemostasis is characterized by vascular constriction and platelet aggregation, degranulation, and fibrin formation (thrombus) [19]. In the inflammatory phase, neutrophils infiltrate the wound site, followed by monocytes and lymphocytes [20]. Re-epithelialization occurs during the proliferation phase, while new blood vessels are formed (angiogenesis), and collagen is synthesized for new extracellular matrix formation [21]. Finally, collagen and the vasculature are remodeled [22]. Many different cell types are involved in the healing process. For wound closure, fibroblasts (and keratinocytes) are essential for extracellular matrix deposition and tissue remodeling. Sequential immigration of neutrophils, macrophages, and lymphocytes supports eradicating infectious agents and mounting antimicrobial immune responses, along with regulating inflammation and orchestrating wound healing phases and cell immigration (Fig. 1).

Chronic wounds are a significant health issue in the western world, causing an estimated $3 billion per year in the US [23,24]. By definition, chronic wounds “failed to progress through the normal stages of healing and therefore enter a state of pathological inflammation” [2]. It is possible that different parts of a wound may be stuck in different healing phases, having lost the ideal synchrony of events [25]. Consequently, healing is delayed and incomplete, resulting in poor anatomical and functional outcomes. Chronic wound etiology is heterogeneous, but most ulcers are caused by ischemia, secondary to diabetes mellitus, venous stasis, and pressure [26]. Additionally, wound healing can be compromised by infection with microorganisms [27]. In healing wounds, inflammatory processes effectively eradicate microbial invasion, while in deteriorated tissues (ischemia, hypoxia, devitalized tissue, and chronic inflammation), this process may be impeded [28]. Consequently, inflammation mediated by, for example, neutrophils cannot be resolved. In routine healing, neutrophil presence is self-limited to 72–96 h after wounding [23], while in chronic wounds, these cells are present in senescent states throughout the (stagnated) healing process [29]. Pharmacological control of inflammation in wound healing remains challenging, however [30].

Unfortunately, to date, there is no generally accepted human-like chronic wound animal model available [31,32], rendering animal experiments challenging to interpret. This applies, for example, to studies addressing the role of bacteria in improper wound healing. While the presence of bacteria is a well-recognized factor in chronic wounds [33], their eradication is not always associated with improved healing responses [34]. Hence, an infection can be both cause and consequence of impeded wound closure.

### 2.2 Gas plasma technology

The term *plasma* is Greek and means “something molded.” Plasma is commonly associated with blood plasma, but the term homonymously also means physical plasma. It was used first in 1879, describing “radiating matter” [35]. In 1928, Langmuir investigated the flow of physical plasma, reminding him of blood plasma flowing through veins [36]. In physics, plasma is described as the fourth state of matter [37]. It is assumed that the majority of visible matter in the universe is in a plasma state. On earth, plasma appears in the form of, e.g., lightning, a*urora borealis*, or fire. Plasmas are also generated artificially for technical applications, such as welding, neon lights, head light systems in cars, light bulbs, and televisions.

Plasma is generated by energizing gas up to a critical point at which electrons dissociate from atoms. Because the resulting ionized gas contains charged particles (electrons and ions), plasmas are conductive while the overall charge remains electrically neutral. The physico-chemical plasma characteristics are complex, dependent on a multitude of parameters as the type and composition of the gas or gas mixture used for plasma generation, applied energy, pressure, and environment. Typical particles being generated include ions, electrons, and reactive atomic and molecular species, which can be charged or neutral. Electric and magnetic fields and light (visible, infrared, ultraviolet) are also generated [38,39].

Different design and engineering concepts of medical gas plasma sources such as jets and dielectric barrier discharges (DBDs) have been extensively reviewed before [40,41]. Marketed gas plasma systems in Europe comply with the guidelines set out by the medical device regulations, are operated at body temperature and atmospheric pressure, and are usually classified as medical devices class IIa [42]. Medical plasma applications are established for a long time in the field of electro surgery, where techniques like argon plasma coagulation (APC) rely on precisely targeted thermal necrotization of tissue to achieve hemostasis (cauterization) or to cut or remove tissue [43,44]. The technical availability of setups for stable and reproducible plasma generation at low temperature under atmospheric conditions, so-called cold atmospheric plasmas (CAP), opened up the new field of plasma medicine, meaning the direct application of physical plasma on or in the human (or animal) body to apply therapeutic effects.

**Fig. 2:**
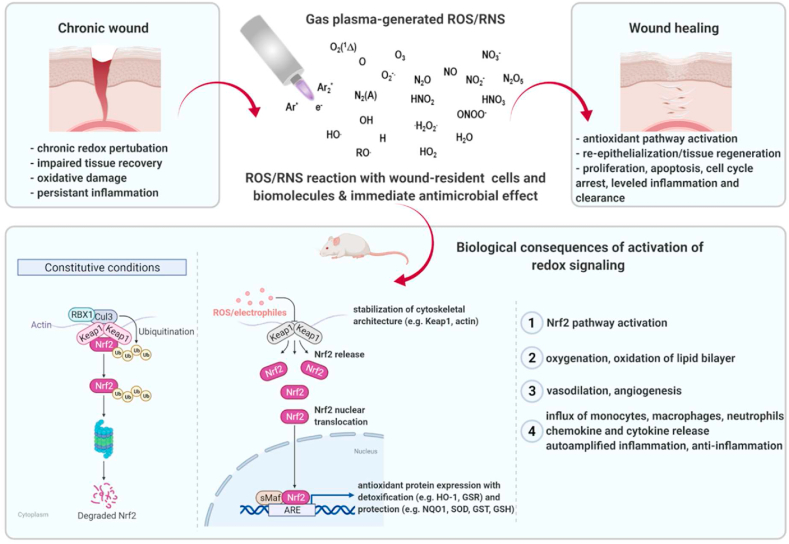
A plasma jet generating several types of ROS/RNS simultaneously and their emittance to the surrounding. The highest concentration of ROS/RNS is within and close to the plasma plume. Among those species generated are, for instance, nitric oxide (NO), peroxynitrite (ONOO^−^), atomic oxygen (O), singlet oxygen (O_2_ (^1^Δ)), superoxide anion (O_2_^−^), hydrogen peroxide (H_2_O_2_), ozone, hydroxyl radicals (OH), nitrogen dioxide (NO_2_), and others. Dimensions are not accurate to scale. Chronic wounds failed to heal. Gas plasma treatment targets chronic wounds and leads to biological consequences during wound healing, including redox signaling, inflammation, tissue regeneration, and protection. In preclinical studies, accelerated healing was associated with improved re-epithelization, angiogenesis, collagen fiber, keratin production, and granulation. Other beneficial effects were observed, including macrophage and neutrophil immigration, chemokine and cytokine release, antioxidant signaling (Nrf2 pathway activation), and proliferative along with wound healing stage-dependent apoptotic (p53 signaling) responses. Created with BioRender.com.

The *kINPen* plasma jet is the best-investigated plasma source from the physics and biomedical point of view [45], and here serves as an example to briefly explain the plasma generation. The plasma is generated by applying a high-frequency alternating voltage to a noble gas, such as argon. The electron flux displays high kinetic energy and is hot. These fast electrons then ionize molecules of the argon feed gas. By contrast, argon molecules (ions) are more heavy-weighted than electrons and hence slower in the electric field, preventing their acceleration. Also, fast electrons inefficiently transfer their kinetic energy to ions which consequently remain cold. As the ion temperature of gas determines its overall temperature, the plasma contains highly energized particles on the one hand without displaying the significant temperature increase that would usually come with their presence on the other: A so-called non-equilibrium plasma. Additional cooling of plasma can be achieved using high feed gas fluxes. The feed gas flux drives the plasma and its charged argon particles to the ambient air containing oxygen and nitrogen, eventually generating ROS/RNS. These are usually mixtures of non-radical (e.g., H_2_O_2_, ozone) and radical species (e.g., hydroxyl radical, nitric oxide; NO) and several other components (Fig. 2) [38]. In other plasma device concepts where atmospheric air is used as working gas for plasma generation, ROS/RNS are also generated, of course, which can be seen as a general characteristic of cold atmospheric plasmas.

The antimicrobial efficiency of gas plasmas started to become appreciated in the 1990s [46] increasingly, and the term “plasma medicine” was subsequently coined [47]. It is important to note that the definition of this term in its essence means the active plasma zone being in direct contact with the target. In other technical setups, such as ozone and NO generators, the gas plasma is ignited within a zone distant from the treatment target, and the generated ozone or NO is subsequently ventilated onto the target, leading to promising results in the 1990s as outlined below. In the following decades, prototype non-medical gas plasma devices were found to inactivate most types of microorganisms, including *Streptococcus mutants* [48], *Staphylococcus aureus* [49], *Pseudomonas aeruginosa* [50], and *Escherichia coli* [51]. More than 100 clinical isolates of wound bacteria, including drug-resistant strains, were eradicated by plasma devices *in vitro* [52]. The development of bacterial resistance was not observed so far [53,54]. It appears plausible that decreasing the bacterial burden with plasma treatment may foster chronic wound healing, provided that there is a treatment time window between the antimicrobial activity and tissue damage caused by gas plasma. Beyond such an antiseptic effectivity of cold atmospheric plasma, there were several early hints from preclinical and clinical research that plasma application can stimulate directly the regeneration of impaired tissue.

While the exact antimicrobial mechanism of action of gas plasma treatment is beyond the scope of this work, it remains noteworthy mentioning that killing bacteria via reactive species is an evolutionary old and conserved and, therefore, effective strategy of higher organisms within phagolysosomes and using chlorinated oxidants [55,56]. However, pinpointing the main antimicrobial and possibly stimulating ROS/RNS in gas plasma is a daunting task. First, hundreds of chemical reactions occur in gas plasmas simultaneously, generating dozens of ROS/RNS in parallel, each having individual spatio-temporal concentrations along with the plasma-ambient air gradients [[57], [58], [59]]. Second, these species have different travel and diffusion distances in the gas phase and the treated tissues due to their diverse reactivity. Third, there is a lack of methods and tools to unambiguously identify the type and quantity of individual species within a complex environment, especially once interacting with tissues. None withstanding, it is evident that many ROS/RNS generated by gas plasma are known to have biological functions [60]. Among those species generated are, for instance, NO, peroxynitrite (ONOO^−^), atomic oxygen, singlet oxygen, O_2_^−^, H_2_O_2_, ozone, hydroxyl radicals, nitrogen dioxide, triplet oxygen, and hydroperoxyl radicals that can lead to secondary species such as organic alkoxyl radicals and organic peroxides [61]. The fundamental insight first summarized by Graves 2012 [31] that ROS/RNS might be the most critical components has opened up the door to redox biology to explain and interpret several biological effects caused by gas plasma.

In summary, medical gas plasma systems are operated at atmospheric pressure and body temperature, expelling ROS/RNS cocktails locally to the exposed site, and treatment effects in tissues and wounds have been demonstrated, as outlined in the subsequent preclinical and clinical studies. For a comprehensive overview of gas plasma devices and operation principles, antimicrobial effects, and *in vitro* findings in eukaryotic cells, the reader is referred to reviews on these topics [[62], [63], [64], [65], [66], [67], [68], [69]].

## 3 Evidence of gas plasma-stimulated wound healing

There is ample evidence of gas plasma-stimulated wound healing in preclinical studies and clinical observations and trials in patients. Moreover, gas plasma exposure of intact skin and its subsequent analysis has been tutorial in better understanding mechanisms of actions.

### 3.1 Preclinical studies on gas plasma treatment in animal wound models

Several animal studies have shown beneficial wound healing-promoting effects of gas plasma exposure of clinically accredited devices and experimental plasma prototypes (Table 1). In two studies, Arndt and colleagues have used an earlier version of the commercially available SteriPlas system in 129 Sv/Ev mice, having received punch biopsies on the dorsum. Increased angiogenesis, wound closure, and macrophage and neutrophil infiltration were observed, along with elevated levels of fibroblast growth factor 2, monocyte-chemoattractant protein 1, collagen type I, and interleukin (IL) 6 [70,71]. Several studies investigated the effects of the atmospheric pressure argon plasma jet *kINPen* on wound healing, which, in principle, follows similar mechanisms observed in wound healing models using the SteriPlas device [72]. Breathnach and colleagues found in gas plasma-treated punch-wounds in rats accelerated wound closure and re-epithelization, together with less fibrosis and more acute inflammation [73]. Studies in ear wounds of SKH1-hr mice re-iterated and extended these results to accelerated healing [74], angiogenesis, re-epithelization, collagen fiber and keratin production, granulation, inflammation, macrophage and neutrophil immigration, and antioxidant (Nrf2) and proliferative (p53) responses [75,76]. The latter study also revealed that H_2_O_2_ exposure did not fully recapitulate the results obtained with gas plasma treatment and that for several findings (re-epithelization; IL-1β, IL-6, TGF-β, Nrf2, HMOX1, NQO1, SOD1, CAT, KGF) differences between short (3 s) and lengthy (20 s) exposure times were identified. This suggests that differential redox regulation depends on the total amount of ROS/RNS deposited to the wound. Notably, the assessment of tissues one year after treatment did not reveal any negative long-term consequences of the gas plasma treatment regarding carcinogenesis and deficiencies of skin architectures [75]. Early after gas plasma treatment (up to day 15), however, regulation of focal adhesion (e.g., fibrillary adhesions and integrin adhesion complexes) and changes in matrix remodeling (e.g., MMPs and TIMPs) were observed in gas plasma-treated wounds [77]. MMP1 and MMP5 have also been reported to increase during singlet oxygen-inducing light conditions [78]. In addition to the safety and molecular aspects mentioned above, gas plasma treatment increased wound tissue oxygenation in superficial and deep layers along with elevated tissue hemoglobin and water indices [79].

**Table 1:** Animal studies on gas plasma-stimulated wound healing. Articles are sorted by year of appearance.

wound size/location	plasma (gas)	main findings in the gas plasma exposure group	Reference
17 × 17 mm skin removal (dorsum)	NO-generator (Air), *no direct plasma application*	Improved angiogenesis, wound healing, granulation, and shortened inflammation in both aseptic and infected (1 × 10^9^*S. aureus*) wounds	Shekhter et al. [105]
6 mm punch (dorsum)	Jet (He/O_2_/N_2_)	Improved epithelization and neovascularization; decreased microbial burden of natural wound flora	Yu et al. [81]
8 mm punch (dorsum)	*SteriPlas* (Ar)	Accelerated wound closure, increased macrophage and neutrophil infiltration, increased MCP-1 and IL-6, more collagen type I	Arndt et al. [71]
10 × 15 mm skin removal (dorsum)	Jet (He)	Accelerated wound closure with intermediate but not low or long gas plasma treatment times	Jacofsky et al. [109]
2 mm punch (dorsum)	Jet (Ar)	Accelerated wound healing and re-epithelization	Nasruddin et al. [85]
2nd ° burn (dorsum)	Jet (Ar/N_2_)	increased healing rates, angiogenesis, blood flow, epithelization, wound contraction, and secondary ROS/RNS in wound tissue	Ngo Thi et al. [82]
5 mm punch (dorsum)	Jet (Ar, Ar/Air)	Accelerated wound closure, better in Ar + Air over Ar, more IL-6 mRNA in Ar + Air-treated wounds	Kim et al. [90]
4 mm punch (dorsum)	Jet (Ar)	Accelerated wound healing and increased myofibroblasts, especially in watered (humidified) wounds	Nasruddin et al. [86]
4 mm punch (dorsum)	Jet (Ar)	Accelerated healing with short and intermediate but not long exposure times	Xu et al. [95]
2 mm punch (dorsum)	Jet (He)	Accelerated wound closure in both non-diabetic and diabetic rats, more acute inflammation and neovascularization	Fathollah et al. [108]
1.5 mm punch (ear)	Jet (He)	Increased angiogenesis and vascularization early after gas plasma exposure	Kim et al. [89]
3 × 20 mm laser (dorsum)	Jet (Ar/N_2_)	Improved wound healing with 3x over 1x exposure; increased blood flow and RNS release into tissue; increased wound strength and laminin; decreased MMP3	Shao et al. [98]
6 mm punch (dorsum)	*SteriPlas* (Ar)	Increased angiogenesis and FGF-2 generation	Arndt et al. [70]
8 mm punch (dorsum)	Jet (He)	Accelerated wound closure, mRNA expression changes (increased IL-6, Nos2, and Ptgs2; decreased Nfκb and Sod1), effects only at day 7 but not at days 3 and 10	Kubinova et al. [100]
4 mm punch (dorsum)	Jet (Ar)	Accelerated wound healing and re-epithelization	Nasruddin et al. [87]
2 × 2 mm skin removal (ear)	*kINPen* jet (Ar)	Accelerated wound healing and angiogenesis	Schmidt et al. [74]
6 mm punch (dorsum)	*kINPen* jet (Ar)	Accelerated wound closure and re-epithelization, less fibrosis and more acute inflammation,	Breathnach et al. [73]
10 mm pressure ulcers (dorsum)	Jet (He)	Accelerated wound closure, increased angiogenesis and re-epithelization as well as inflammation, higher force resistance and elastic stiffness	Chatraie et al. [99]
8 mm punch (dorsum)	Jet (Ar)	Accelerated wound closure in both healthy and Type I and Type II diabetic rats; improved re-epithelization and fewer neutrophils and T cells in diabetic wounds at late stages; increased SOD and catalase as well as GPx in treated tissues	Cheng et al. [107]
6 mm punch (dorsum)	Jet (He)	Accelerated wound healing across several exposure durations	Shahbazi Rad et al. [94]
17 × 17mm skin removal (dorsum)	NO-generator (Air), *no direct plasma application*	Accelerated wound healing; decreased long-term inflammation; improved collagenI/collagenIII ratio	Shekhter et al. [104]
4 mm punch (dorsum)	Jet (Ar)	Lack of beneficial effect of combination with hydrocolloid dressings and medical honey	Wahyuningtyas et al. [88]
4 mm punch (dorsum)	Jet (Ar)	Long exposure of direct contact-style jet treatment heated the tissue (>50 °C) and was highly detrimental for wound healing; longer distances promoted wound healing and epithelization	Darmawati et al. [83]
4 mm punch (dorsum)	Jet (Ar/Air)	Accelerated healing with dual-frequency over single-frequency and 2 over 1, 3, and 4 treatment cycles	Lee et al. [92]
2 × 2 mm skin removal (ear)	*kINPen* jet (Ar)	Accelerated wound healing and angiogenesis as well as re-epithelization; increased collagen fibers, keratin production, Nrf2 response, p53 activation, macrophage infiltration, inflammation, and granulation	Schmidt et al. [76]
13 × 13 mm skin removal (dorsum)	Jet (He)	Accelerated wound healing, angiogenesis, epithelization, and wound contraction; collagen unchanged; TNFα and IL-1β but not IL-10 increased	Zhang et al. [102]
17 mm punch (dorsum)	Jet (He; He/Ar)	Accelerated wound healing and granulation tissue formation; He/Ar performed better than He gas plasma; short gas plasma treatment times performed best	Lou et al. [93]
40 × 40 mm (dorsum)	Jet (He)	Accelerated wound healing and increased angiogenesis	Martines et al. [80]
6 mm punch (dorsum)	Jet (He, He/O_2_)	Accelerated wound healing and highest VEGF and bFGF with He/0.1% O_2_ over He and He/1% O_2_	Pan et al. [91]
6 mm punch (dorsum)	DBD, Jet (He; Ar)	Improved wound healing with DBD but better wound healing with Ar and He gas plasma jet treatment	Shahbazi Rad et al. [96]
2nd ° burn (dorsum)	Jet (Ar)	Increased early leukocyte influx, MPO release, free thiols, and angiogenesis	Souza et al. [101]
10 × 10 mm skin removal (dorsum)	Jet (He)	Accelerated wound healing; less scar formation, TGF-β/pSmad2/3/αSMA positive cells, and collagen deposition	Wang et al. [84]
2 × 2 mm skin removal (ear)	*kINPen* jet (Ar)	Changes in matrix remodeling and focal adhesion complex; elevated microcirculation	Schmidt et al. [77]
2 × 2 mm skin removal (ear)	*kINPen* jet (Ar)	Increased wound tissue oxygenation in superficial and deep layers as well as increased tissue hemoglobin index, increased tissue water index at late time points	Schmidt et al. [79]

Using experimental plasma sources, several studies confirmed the wound healing-promoting properties of gas plasma exposure. Apart from one report showing accelerated wound healing in Bergamasca sheep [80], all studies used rodent models. The first report was in 2011, showing beneficial effects of gas plasma exposure on wound size reduction, granulation, epithelization, and neovascularization in mice [81]. A study three years later confirmed these results revealed extended early inflammation, elevated cellular proliferation, and epithelization, and increased NO tissue deposition accompanied by improved angiogenesis [82]. It is also clear that the gas plasma system must be operated at body temperature, as higher temperatures were detrimental to wound healing in mice compared to an electric mode generating a ‘cold’ argon plasma [83]. Apart from beneficial wound healing responses, recent work reported reduced scar formation in mice [84]. Nasruddin and colleagues not only identified improved healing responses in Balb/c mice using an Ar plasma jet [85] but also determined in a follow-up study that moisturized wounds markedly increase the extent of gas plasma-promoted wound healing and myofibroblast formation [86]. However, combining with other methods, for example, medical honey, was less successful [87,88]. Such wound pre-conditioning has otherwise received only little attention in plasma medicine wound studies so far.

In ear-wounded SKH1-hr mice, a helium plasma jet accelerated angiogenesis and vascularization [89]. In the same mouse strain, another study identified low air admixtures into an argon plasma showing improved wound healing compared to the lack of air admixture [90]. Even more intriguingly, the type of feed gas and the percentage of admixture affect wound healing parameters. It was identified that He/O_2_ (0.1%) plasma showed favorable wound healing responses than He and He/O_2_ (1%) conditions [91]. Investigating another plasma parameter, Lee and colleagues found that the excitation frequency of the gas plasma device affected the extent of wound healing promotion [92]. The importance of the gas composition was re-iterated in wounded Sprague Dawley rats, showing accelerated healing and granulation tissue formation with He/Ar over He plasma mixtures and short over long exposure times [93]. In addition, the treatment duration of gas plasma exposure [94,95] and the type of source (DBD vs. jet) [96] was found to be critical for improved wound healing in Balb/c mice. Moreover, He plasma needle-stimulated wound healing was attributed to RNS (e.g., NO), whereas Ar plasma predominantly caused blood coagulation [97]. These studies suggest that the ROS/RNS amounts and mixtures of gas plasma systems can be optimized for targeting therapies by a differential enrichment of some species over others, while the absolute exposure time also plays a significant role in healing responses. The number of gas plasma exposure cycles also affects wound healing, as three treatments outperformed single treatments in terms of re-epithelization, NO deposition into wound tissue, blood flow, laminin production, and wound strength in laser-induced wounds in the skin of C57/BL6 mice [98]. In a rat model of dorsal pressure ulcers, Chatraie and colleagues found accelerated wound closure, angiogenesis, re-epithelization, inflammation, force resistance, and elastic stiffness using a helium plasma jet [99]. In Wistar rats receiving regular punch-biopsy wounds and helium plasma jet treatment, accelerated healing and increased *IL-6* and *NOS2* were identified at day 7 post wounding [100]. Another study using burn wounds in Wistar rats focused on the inflammatory consequences of gas plasma exposure [101]. The number of leukocytes was increased at d2, similar on days 7 and 14, and decreased on d21 post wounding. Free thiols were maximal at d2, myeloperoxidase (MPO) and VEGF were elevated on only d7, IL-17 cells were increased only on d14, and the collagenI/collagenIII ratio was the highest at d21. Throughout d2-d21, IL-10 was decreased and blood vessel formation was increased, with TGF-β being increased from d2-d14. Together with another report showing elevated levels of TNF-α and IL-1β [102], these data suggest that gas plasma exposure enhances inflammatory processes early on. Paradoxically, this simultaneously seems to enhance antioxidant and anti-inflammatory processes, ultimately accelerating angiogenesis, tissue formation, and matrix remodeling [101]. In this view, it can be hypothesized that gas plasma exposure markedly increases existing biological tissue formation pathways that are intertwined with both inflammatory and redox signaling pathways. This is underlined by improved healing responses in aseptic and infected wounds in rats using an air-plasma NO donor device [[103], [104], [105]] that together with NADPH oxidase (NOX)-derived O_2_^−^ might also had amplified ONOO^−^ in the wound microenvironment. Again, it should be stressed that this device is not directly categorized as plasma medicine device, since the gas plasma is not directly in contact with the target. Nevertheless, it emphasizes the importance and potential that plasma technology is having in wound healing specifically and applied redox medicine in general.

Venous insufficiencies and neuropathy make diabetic patients especially prone to defective wound healing [106]. Healthy rats or rats conditioned with type I or type II diabetes received punch-biopsy wounds at the dorsum and showed improved wound healing responses following exposure to an argon plasma jet [107]. This was accompanied by elevated re-epithelization and SOD, CAT, and GPx expression. A less extensive study confirmed the beneficial effects of gas plasma in healing responses in diabetic rat wounds using a helium plasma jet [108]. In diabetic BKS.CG mice, promotion of wound healing was also found but was dependent on the gas plasma exposure time [109]. A complementary study in diabetic db/db mice found a dependence of improved wound healing on the gas plasma condition, which was associated with increased VEGF and bFGF expression levels [91].

Summarizing findings from these animal studies, it is concluded that gas plasma-stimulated wound healing is present in both wild type and diabetic rodents and independent of the vertebrate species investigated or the type of acute wound (punch biopsy, surgical removal of epidermis and dermis, laser-induced wound, heat-induced burn) so far. As these studies did not study experimentally infected wounds, effects of gas plasma-derived ROS/RNS on the wound cell types and microenvironment are hypothesized to be the primary mechanism of action in these models, putting this field into the heart of redox medicine [69]. Strikingly, the gas plasma treatment process was critical in evoking improved wound healing. This included the exposure time, the feed gas admixture and subsequent ROS/RNS cocktail used, the excitation frequency, the number of treatment cycles, and the type of device in use. In general, most studies used plasma jets rather than flat-shaped DBD systems, as the former benefit from improved penetration of micro-cavities, which are abundant in wounded tissues. Biological consequences of gas plasma and exposure were found across all wound healing phases (Fig. 2). ROS/RNS are ancient signaling molecules, being the first to be released upon tissue injury and initially evoking inflammation and leukocyte chemoattraction [110]. Both were also found in the rodent wound models upon gas plasma exposure, which was followed by accelerated antioxidant defense responses, growth factor release, angiogenesis, re-epithelization, and remodeling. Nevertheless, it needs to be emphasized that the congruency of studies was little in terms of device and feed gas used, gas plasma exposure time and the number of treatment cycles, time points of wound tissue analysis, wound and rodent model employed, and biological effectors analyzed. While similar trends are observable across all studies, more standardization and harmonization of study designs are needed to allow more cross-fertilization. This is needed to identify key wound effector pathways responsible for the accelerated healing responses, and optimized gas plasma ROS/RNS cocktails with concurrent identification and technical enrichment of reactive species critical for driving wound closure efficiently. Key conclusions are summarized in box 1.

Box 1 **Conclusions from animal studies on gas plasma-stimulated wound healing.**

Conclusions from animal studies on gas plasma-stimulated wound healing, which are

i. Found in non-infected wounds, suggesting a role of redox-biological processes independent of antimicrobial action,
ii. Apparent in skin damaged via punch biopsies, incision, and heat (burn),
iii. Present in both wild type and diabetic animals,
iv. Identified across several species, such as mice, rats, and sheep,
v. Dependent on several gas plasma treatment parameters, such as exposure time, gas mixture and subsequent ROS/RNS cocktail, excitation frequency, number of exposures, and device type,
vi. Associated with increased inflammation, leukocyte infiltration, and generation of inflammatory mediators (chemokines and cytokines) followed by accelerated antioxidant defense responses, growth factor release, angiogenesis, tissue oxygenation, re-epithelization, and tissue remodeling,
vii. A function of the time point of the investigation, wound healing phase, and study design,
viii. Particularly realized using gas plasma jet systems over DBD systems.

Alt-text: Box 1

### 3.2 Gas plasma exposure of human wounds

For the treatment of experimental or clinical wounds in volunteers and patients, relatively few devices have been tested so far. This owes to the fact that such studies are essential for plasma devices envisioned to be accredited as a medical device for clinical (not cosmetic) application and sold for this purpose. Only a handful of research environments have taken this step so far (Table 2). It is noteworthy that several studies on gas plasma-generated NO therapy have been performed in Russia starting in the late 1990s. A device named Plasotron/Plazon was used as a NO-source, and promotion of wound healing was reported in 68 patients [103] and later in 318 patients [111] and a case study in 2018 [112]. Using another unnamed plasma device, beneficial effects on wound healing were subsequently reported in 113 patients by another group in 2001 [113]. Unfortunately, the study descriptions are not sufficient to retrieve further information. As outlined above, such NO generators do not put the active plasma zone in direct contact with the target and are therefore not classified as medical plasma devices. Nevertheless, these devices reflect the potential of this technology in therapeutic redox biology and medicine.

**Table 2:** Gas plasma application in experimental and clinical patient wounds. Articles are sorted by year of appearance. random. = randomized.

patients	condition	plasma device	main finding in gas plasma-treated wounds	reference
68 (case collection)	Chronic wounds	NO-generator (Air); 5–12 s every or every other day, *no direct plasma application*	Improved tissue hemodynamics and healing responses	Shekhter et al. [103]
36 (random.)	Chronic wounds	*SteriPlas* (Ar); 5 min daily (except weekends) for days to weeks	Well tolerated; significantly reduced bacterial load	Isbary et al. [114]
24 (random.)	Chronic wounds	*SteriPlas* (Ar); 2 min daily (except weekends) for days to weeks	Well tolerated; significantly reduced bacterial load, regardless of species	Isbary et al. [115]
5 (volunteers)	Laser-induced acute wounds	*kINPen* (Ar); 10 s, 30 s, 10 s once for 3 days, vs. control	Well tolerated; repeated short-term (10s) exposure provoked best healing responses	Metelmann et al. [120]
40 (random.)	Acute skin graft wounds	*SteriPlas* (Ar); 2min daily (except weekends) for days to weeks	Well tolerated; significantly improved healing; positive effects on re-epithelization, few fibrin layers, and blood crusts	Heinlin et al. [117]
70 (random.)	Chronic wounds	*SteriPlas* (Ar); 3–7 min daily (except weekends) for days to weeks	Well tolerated; non-significant wound size reduction but significant wound width decrease	Isbary et al. [116]
5 (volunteers)	Laser-induced acute wounds	*kINPen* (Ar); 10 s, 30 s, 10s once for 3 days, vs. control	Increased inflammation at day 6; less post-traumatic disorders at day 180; no long-term side effects at day 360	Metelmann et al. [121]
6 (volunteers)	Vacuum-generated acute wounds	*kINPen* (Ar); 60 s single application	Well tolerated; significantly increased healing	Vandersee et al. [123]
14 (random.)	Chronic venous leg ulcers	*PlasmaDerm* (DBD); 2 × 45 s/cm^2^ 3x/week for 8 weeks	Well tolerated; significantly reduced bacterial load; wound size reduction by trend	Brehmer et al. [118]
34 (case collection)	Chronic wounds	*kINPen* (Ar); 60 s/cm^2^	Well tolerated; significantly reduced wound exudation and microbial burden, especially in combination with antiseptics; antiseptics but not gas plasma notably changed wound microbiome	Klebes et al. [126]
16 (case collection)	Chronic leg ulcers	*kINPen* (Ar); 60 s/cm^2^ 3x per week over two weeks	Well tolerated; significantly higher microbial reduction; complete removal of *P. aeruginosa*	Ulrich et al. [125]
50 (random.)	Chronic pressure ulcers	Microbeam (DBD, Ar); 1 min/cm^2^ 1/week for 8 weeks	Well tolerated; significantly improved healing, reduced exudate, reduced microbial contamination	Chuangsuwanich [131]
1 (case report)	Acute 2nd-degree burn	Jet (He); 3 min per wound (79 cm^2^, 9 cm^2^) 2x/day once	Well tolerated; no increase in inflammation, but thick scab was present early already	Betancourt-Angeles et al. [134]
4 (case collection)	Chronic skin graft forearm wounds	*kINPen* (Ar); 30–60 s/cm^2^ single application	Well tolerated; no increase of inflammation or infection; complete healing of all wounds	Hartwig et al. [124]
6 (case collection)	Chronic wounds after craniomaxillofacial surgery	*PlasmaDerm* (DBD); 90 s/cm^2^ twice within 1 week	Well tolerated; no increase of inflammation or infection; complete healing of all wounds	Hartwig et al. [119]
4 (case collection)	Chronic diabetic foot ulcers	Jet; 1 min every second day (7x in total)	Elevated levels of TGF-β, EGF, and KGF in wounds	Naderi et al. [135]
1 (case report)	Diabetic foot ulcer	Jet (He);	Complete healing of a chronic ulcer	López-Callejas et al. [133]
1 (case report)	Chronic wound	NO-generator (Air); 5 min and a total of 33 treatments, *no direct plasma application*	2.5 years therapy-resistant wound completely healed	Pekshev et al. [112]
32 (case collection)	Chronic ulcers	DBD (He); 30 s/cm^2^ 1/day until patient suspension	Well tolerated; ulcer healing rates were 100% (neuropathic), 75% (chronic), and 59% (mixed)	González-Mendoza et al. [132]
12 (volunteers)	Laser-induced wounds	*kINPen* (Ar); 60 s/cm^2^ single application	In trend improvement of wound healing; no effect on melanin production	Nishijima et al. [122]
7 (case collection)	Superficial skin wounds: Pyoderma gangrenosum (2), trauma (2), genital warts (1), diabetic foot ulcer (1), chronic eczema (1)	Multi-electrode DBD; 2–8 applications	Well tolerated; complete remission and wound healing achieved in all patients; no side effects observed	Gao et al. [136]
44 (random.)	Chronic diabetic foot ulcers	Jet (He); 5 min 3x/week for 3 weeks	Well tolerated, decreased IL-1, IL-8, IFNγ, TNF-α independent of plasma; less antimicrobial burden; clinical benefit modest	Amini et al. [129]
44 (random.)	Chronic diabetic foot ulcers	Jet (He); 5 min/3x per week for 3 consecutive weeks	Significantly improved wound healing and short-term antimicrobial effects	Mirpour et al. [130]
45 (random., multicentric)	Chronic diabetic foot ulcers	*kINPen* (Ar); 30 s/cm^2^ per day for 5 consecutive days followed by 3x every 2nd day	Well tolerated; improved healing speed and wound reduction independent from background infection	Stratmann et al. [127]
10 (controlled pilot study)	Split skin graft donor sites	*PlasmaDerm*-Dress disposable plasma patch; 90s per wound 3x/day for 7 consecutive days	Well tolerated; significantly improved tissue oxygenation, hemoglobin wound content, and tissue water index; significantly reduced wound pain reduction	Van Welzen et al. [137]

More detailed studies are available on the clinical experience of the accredited medical gas plasma devices in Europe. In the first randomized, prospective clinical trial in plasma medicine, earlier versions of the *SteriPlas* device significantly reduced microbial burden in chronic wounds in 36 patients [114], independent of plasma device modifications and the type of microorganism in 24 patients [115]. For some wound parameters, a significant reduction was observed in a follow-up trial in 70 patients [116]. The *SteriPlas* system was also successfully used to promote the healing of skin graft donor sites in a randomized placebo-controlled trial in 40 patients [117]. A significant reduction in bacterial load and wound size was observed in another randomized prospective clinical trial in 14 patients using the PlasmaDerm device [118]. The device was also suitable for treating surgical wounds in a study of six patients [119]. For the plasma jet *kINPen*, several reports on clinically relevant wound healing are available. In experimental, laser-induced acute wounds on the forearms of five volunteers, *kINPen* plasma treatment promoted objective healing responses [120] without adverse long-term effects such as scaring [121]. Using a similar study setup in 12 patients, these results were confirmed by Nishijima and colleagues, who moreover observed neither an increase nor decrease of melanin production [122]. Significantly improved healing was also observed in *kINPen* treated vacuum-generated wounds in volunteers [123]. A small study of four patients with chronic skin graft forearm wounds outlined the possibility of using the *kINPen* plasma to promote healing in this condition [124]. Chronic leg ulcer treatment with the gas plasma jet led to a significantly higher microbial reduction in a case series of 16 patients [125]. In a more extensive study in 34 patients suffering from insufficient wound healing, *kINPen* plasma treatment significantly reduced wound exudation and microbial burden [126]. Interestingly, antiseptics but not plasma treatment changed the wound microbiome, suggesting plasma to perform equally well across different bacterial species. Recently, a randomized prospective clinical trial in patients suffering from diabetic foot ulcers re-iterated the wound-healing promoting properties of the *kINPen* plasma treatment [127]. Strikingly, plasma exposure was performed in addition to wound antiseptics, suggesting the mode of action of plasma treatment to be independent or at least less dependent on its antimicrobial efficacy. Moelleken and colleagues used the *SteriPlas* device in a randomized clinical pilot study with 37 patients with therapy-refractory chronic wounds. A significant reduction of wound area was detectable in the plasma-treated groups but not in the non-treated control group. Interestingly, there was no difference in wound healing in the group that was plasma treated three times per week compared to that group with a single treatment per week. Because patients undergoing systemic antibiotic treatment or topical antimicrobial treatment where excluded, a differentiation between antiseptic plasma effect and direct stimulation of tissue regeneration was not possible [128].

Clinical observations have also been reported for other plasma sources that have not (yet) received accreditation in the western countries. In a randomized trial in Iran with 44 patients suffering from diabetic foot ulcers, helium plasma jet treatment led to decreased production of IL-1, IL-8, IFN-γ, and TNF-α as well as less antimicrobial burden, while clinical benefit was relatively modest [129]. This suggests the plasma treatment to decrease inflammation, possibly as a result of decreased infection. Using a different type of helium plasma jet, a similar randomized study was performed in 44 patients that, by contrast, identified significantly improved healing responses [130]. Notably, the authors found potent antimicrobial efficacy that, however, was only transient. These observations have also been made for the *kINPen* (unpublished observation). Significantly improved healing and reduced exudate and microbial contamination were also found with a microbeam DBD (BIO-PLASMA CELL modulation) in a randomized trial with 50 patients in Thailand. In contrast, the microbial composition in the wound did not change [131]. In addition, a case collection of 32 patients with chronic ulcers was reported to benefit from helium DBD plasma treatment to a lesser or greater extent, depending on ulcer type [132]. Besides other helium plasma jet case reports providing data on the safe application in wound healing [133,134], an interesting mechanistic study using 2D gel electrophoresis of wound exudates before and after plasma treatment in 4 patients, elevated levels of TGF-β, EGF, and KGF were found in plasma conditions [135]. A case report series from seven patients suffering from long-standing superficial skin wounds of several etiologies used a six-electrode-array experimental DBD device and achieved complete remission and wound healing in all patients after two to eight applications [136]. The latest concepts in plasma technology involve utilizing disposable plasters or dressings that plasma technology integrated into the products that are connected or on-demand connectable to a power supply to control the gas plasma exposure and timing. The benefit of this approach is that gas plasma application can be applied several times while the dressing remains on the wound without time-consuming manual intervention needed by medical staff. Due to the disposable nature of such devices, sterility can be guaranteed, but it needs to be elaborated for future certified products, which are so far not available, whether this method is also cost-effective compared to other approaches. Only recently, a first controlled pilot study on the response and tolerability of a novel DBD-based wound dressing has been published [137]. Ten patients with split skin graft donor sites were enrolled. The treatment significantly improved tissue oxygenation, hemoglobin content, and tissue water index in the wounds, accompanied by decreased wound pain and high tolerability of the procedure without any side effects. The *PlasmaDerm-Dress* device (Cinogy GmbH) is currently pursuing certification of that device. Other companies, such as ColdPlasmaTech GmbH (Greifswald, Germany), are also working on such technologies but published reports on the safety and efficacy of their plasma patch are not available so far. An overview of devices and technologies can be found in more medically-focused reviews [42,[138], [139], [140]].

## 4 Mechanisms of gas plasma-stimulated wound healing

**Fig. 3:**
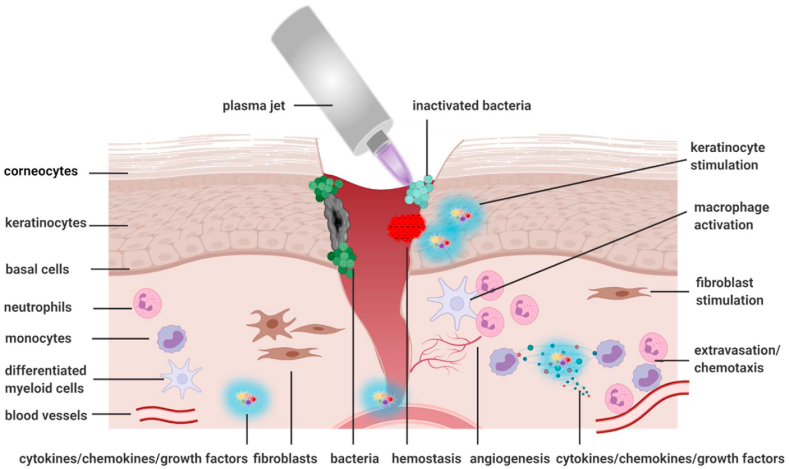
Wound-relevant targets potentially affected by gas plasma treatment within and near the wound bed. Many different cell types, such as epidermal keratinocytes, dermal fibroblasts, and immune cells, are involved in the healing process. Microbial growth is reduced immediately following wound treatment. Gas plasma induces keratinocyte and fibroblast stimulation, macrophage activation, and infiltration and migration of neutrophils and lymphocytes along a chemotactic gradient towards the site of tissue damage. Created with BioRender.com.

Compelling preclinical and clinical evidence underlines the favorable healing responses of gas plasma-stimulated wounds. In the following, several mechanisms of gas plasma-stimulated wound healing are outlined. It comes as no surprise that neither for wound healing nor the action of gas plasmas, a single agent or mechanism can be singled out. Wound therapeutic needs depend on the context (see next section) and healing phase, making a set of mediators *a priori* likely [141,142]. Against this complexity, gas plasma systems are *per se* of multicomponent nature, and accurately isolating individual components without any actions of others or even separating some types of ROS/RNS from others in the plasma gas phase is technically impossible. Hence, the plasma gas phase and ROS/RNS trajectories have been characterized very well on the one end, while on the other end, the biological effects in skin and tissues have been investigated in dozen studies. Less is known for the mechanisms in between, partly because of the short time scales ROS/RNS react with their targets and each other, partly because the field of redox biology currently lacks the tools to distinguish the contribution of different types of ROS/RNS appearing simultaneously on a quantitative scale [67]. Notwithstanding, a set of mechanisms and hypotheses is proposed that contribute to gas plasma-stimulated wound healing (Fig. 2, Fig. 3). Instead of a single mechanism, we here propose several to act in concert to promote healing. From the cited studies, it can be concluded that such mechanisms are independent of the species, i.e., gas plasma-derived ROS/RNS mixtures drive an evolutionary older program of tissue regeneration.

For many years, the antimicrobial activity of gas plasma exposure was hypothesized to be mainly responsible for improved healing. Yet, studies in sterile animal wounds (Table 1) and patients receiving gas plasma therapy combined with antiseptics [127] suggest a mode of action independent of microbicide activity. This does not question the critical role of infection in compromised wound healing, especially in immunosuppressed patients and with AMR (antimicrobial resistance) strains. Vice versa, the presence of microorganisms is not necessarily the cause of defective wound healing but can also be its consequence. In line with the findings of many animal studies and a recent randomized controlled clinical trial [127], it follows that gas plasma therapy spurs the endogenous wound healing capacity beyond its demonstrated antimicrobial activity. This is supported by the observation that microbial growth is often reduced immediately following gas plasma wound treatment, returning to the elevated baseline values if investigated one day later and implying that the gas plasma-reduced microbial load may not be durable.

A factor likely contributing to gas plasma-assisted wound healing is the immediate tissue responses observed using hyperspectral imaging. In gas plasma-treated ear wounds in mice, increased superficial and deep tissue microcirculation, tissue oxygenation, and water content was observed immediately following exposure [77]. Similar findings were reported in gas plasma-treated patient wounds [143] and intact human skin [[144], [145], [146]]. Several mechanisms may account for these findings. Plasma jets such as the *kINPen* are rich sources of NO [147,148], which is known for its arterial vasodilation to subsequently increase blood flow. This could be even enhanced by the intrinsic thermal energy of plasma being at or slightly above body temperature (37–40 °C) and therefore warming the treated target (skin: approx. 30 °C), which is known to spur endogenous NO production [149] to augment blood flow further. Increased microcirculation enhances the transport of nutrients, the transmigration of new and unprimed leukocytes, and eventually also of oxygen to counteract hypoxia, one of the key drivers of wound ulceration [150]. Hypoxia is also corrected by increased angiogenesis, as observed in several animal models following gas plasma therapy that showed continuously increased vascularization and tissue oxygenation [70,76,82]. Such mechanisms are in line with therapies projected to address problematic wound hypoxia one decade ago [11].

Two decades ago, it was proposed that wound healing is subject to redox control and that redox-based strategies can be used to treat chronic wounds [8]. Today, there is ample evidence that gas plasma therapy fulfills this criterion. *In vitro* [151] and *in vivo* evidence [76] found a solid stimulation of the antioxidant nuclear transcription factor Nrf2, which was recently described to be a valuable target for pharmacological activation and wound healing promotion [152]. Over three months, repeatedly gas plasma-treated keratinocytes *in vitro* continuously express increased levels of heat-shock protein 27 to counteract the oxidative stress [153]. In wounded murine skin, scab formation occurs, and gas plasma treatment of the wound margins around the scab promoted wound healing below the scab, arguing for redox signaling components that relayed ROS/RNS detection to remote tissue sites [71]. In intact murine skin, gas plasma exposure led to elevated cellular proliferation and Nrf2 and catalase expression [154]. In a rat burn wound model, gas plasma treatment increased the levels of free thiols in the wound tissue, arguing for the Nrf2 pathway to be active upon ROS/RNS exposure and signaling [155]. *Ex vivo* gas plasma-treated human skin showed enhanced proliferation, but for longer exposure times, proliferation decreased [156], being in congruence with the oxidative eustress and distress paradigm proposed by Helmut Sies [157]. Such dual and hormetic role of gas plasma-derived ROS/RNS acting as signaling agents at low concentrations and damaging agents at higher concentrations is well observed in a healing promotion effect of short (3 s) over intermediate (20 s) exposure times in ear-wounds in mice [76]. Finally, it should be re-emphasized that gas plasma-derived ROS/RNS such as O_2_^−^, NO, ONOO^−^, hypochlorous acid, H_2_O_2_, nitrite, nitrate, hydroxyl radical, and singlet oxygen are also part of physiological ROS/RNS generation by NOX, MPO, and NOS, and therefore capitalize on existing – even if partially less explored – redox signaling pathways.

However, the *in vivo* models reflect the clinical situation only to a limited extend. They have no severe underlying disease or excessive infection that generates wound chronification resembling the situation found in patients. In animal models, wound healing is sometimes somewhat impeded but functional, while in patients, healing often is completely halted. Hence, the question is not how to spur wound healing but how to set it in motion in the first place, often referred to as turning a chronic wound into an acute wound. Multifactorial processes are thought to cause the steady-state wound, such as hypoxia, infection, and senescent cells [158]. Especially senescent fibroblasts [159] and neutrophils [160] are associated with impaired healing, and apoptosis induction via gas plasma-induced oxidative distress at modest scales might be a mechanism of action and testable hypothesis in clinical plasma research. Neutrophils and their products, including neutrophil extracellular traps (NETs), are readily present in acute wounds [161] and promote pathogen clearance and inflammation. Gas plasma exposure promotes NET formation [162] and integrin and selectin expression [163] *in vitro*. Albeit excessive NET formation is associated with chronic wounds [164], it remains to be established whether such NETs are the cause or consequence of hampered healing and whether *de novo* NET formation from dying neutrophils might outcompete the negative consequences of live, senescent neutrophils. Many wound therapies generally target inflammatory processes. With the many wound cell types, dozens of chemokines and cytokines, and their interplay within the different healing phases, straightforward solutions for controlling inflammation towards optimum healing have not been identified yet [165]. Several chemokines and cytokines are known to hallmark chronic wounds [166,167], and their generation and release are generally a consequence of signaling. Hence, while some therapeutic strategies aim at supplementing or removing distinct agents from the wound bed with moderate to good success [[168], [169], [170]], targeted wound therapy would address the cause of such pathological release. We hypothesize macrophages to be a primary cell type responding to gas plasma-derived ROS/RNS. Proper healing depends on macrophages orchestrating appropriate inflammation levels that first allow removing infectious agents, and second promotes keratinocyte migration and epithelial-to-mesenchymal transition for re-epithelization of the wound bed, followed by matrix reorganization [171]. A testable hypothesis is a solid gas plasma-induced Nrf2 activation in wound-macrophages, leading to pro-healing M2 polarization, anti-inflammatory cytokine release, increased angiogenesis, and improved matrix remodeling [172].

Finally, chronic wounds are subject to repeated micro-injuries and bleeding. Especially during debridement, a routine procedure to clean the wound of necrotic and excess material, bleeding occurs. Gas plasma treatment is strongly hemostatic, as shown in liver injury in mice, and such coagulation was dependent on platelet activation [173]. The mechanism of action was recently unraveled in human blood platelets that are strongly activated via gas plasma-mediated hemolysis [174]. Platelet activation leads to a series of wound-healing promoting events via, e.g., secretion of factors such as platelet-derived growth factor and epidermal growth factor [175], which has been thoroughly investigated as biological therapy in clinical trials for the promotion of wound healing [176]. Interestingly, autologous platelet gel therapy has emerged as therapy for wound healing, hemostasis, and local infection control [177]. Electric pulse stimulation has been suggested to activate platelets in wounds *in situ* [178], and gas plasma treatment may also do so.

## 5 Potential side effects of gas plasma-stimulated wound healing

Novel therapies have to show promising clinical efficacy while demonstrating no or acceptable levels of side effects. It was reported that the gas plasma treatment was well tolerated for all plasma sources and studies without noticing any SAEs (serious adverse events). As a conclusion from hundreds of clinical gas plasma therapies, the technology is considered safe [42,179], provided appropriate safety tests and accreditation has been performed. As an orientation, a catalog has been published outlining guidelines on safety testing [180]. Key conclusions are summarized in box 2.

Box 2 **Conclusions from human studies on gas plasma-stimulated wound healing.**

Conclusions from human studies on gas plasma-stimulated wound healing, which is

i. Independent of the technology used (jet vs. DBD),
ii. Independent of the feed gas used (argon vs. helium),
iii. Accompanied by decreased microbial burden,
iv. Not changing the composition of the microbial community,
v. reported to decrease wound size modestly to significantly,
vi. Found to be effective across several types of wounds and etiologies,
vii. Applied adjuvant to and not instead of standard-of-care,
viii. Mainly applied multiple times.

Alt-text: Box 2

Several gas plasma devices have been investigated for potential side effects regarding genotoxicity. For the SteriPlas device, no genotoxicity was observed using the HPRT (hypoxanthine-guanine phosphoribosyltransferase) assay according to the OECD protocol [181]. A similar lack of genotoxicity in the HGPRT assay was observed for the *kINPen* [182]. Another OECD-accredited genotoxicity reference assay, the cytokinesis-block micronucleus assay, did not identify micronuclei in *kINPen*-treated cells when compared against positive controls such as ionizing radiation, UV radiation, and genotoxic drugs [183,184]. As mentioned above, no tumor formation was observed in mice after one year – translating to 60 human-equivalent years – after repeated gas plasma treatment of wounds [75]. Also, the healed wounds were void of skin abnormalities, such as hypertrophic scar formation. In patients, a one-year and five-year follow-up of gas plasma-treated wounds confirmed the lack of abnormal healing responses as investigated using hyperspectral imaging and confocal laser scanning microscopy [121,185]. In patients, other side-effects such as extensive pain or discomfort during gas plasma exposure have not been observed [137,[186], [187], [188]]. It would be expected that too few gas plasma applications may result in the non-sufficient promotion of healing responses, while too many applications might cause undesired toxicity. However, gas plasma wound treatment of three times per day [137], three times per week [127,189], and one time per week [128] all led to improved wound healing, suggesting a broad window of application of this technology in chronic wounds. Contraindications may vary between devices and involve, for instance, electroconductive implants, heart failure during the last six months, and pregnancy. The main indication for gas plasma therapy remains the treatment of chronic wounds. For other skin conditions without wounding, such as pruritus or florid vasculitis, reports are absent or did not show efficacy of gas plasma therapy [190]. For pyoderma gangrenosum, however, three case reports concluded a promising role of gas plasma in targeting this disease [136,189].

## 6 Current challenges in gas plasma-stimulated wound healing

Current challenges in gas plasma-stimulated wound healing are two-fold. From a scientific point of view, this ‘positive’ control for wound healing provides an ideal test system to better understand the needs and molecular effectors of proper wound healing. Here, wound exudate sampling offers a compelling window for analyzing the mediators at work across the different stages and before and after plasma treatment. Mass spectrometry is a resource-demanding versatile technology to monitor wound proteins in this regard [161,191]. Less global but more practical is utilizing multiplex chemokine and cytokine analysis. Both approaches require standardized wound exudate collection procedures, which are yet to be determined. Ideally, this would be accompanied by wound biopsies, but this process is not encouraged from a clinical perspective to not further disturb the wound bed, which is why relatively few studies are available in this regard.

**Fig. 4:**
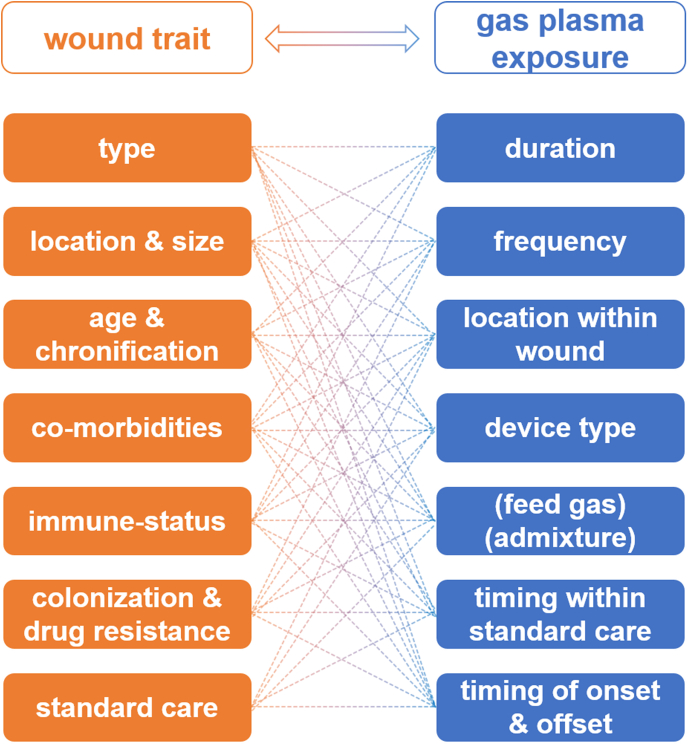
The interdependency of wound characteristics and unknown or non-standardized gas plasma therapeutic schemes, necessitating additional clinical studies to facilitate optimized gas plasma wound therapy.

From a practical, clinical point of view, the challenges of gas plasma application in wound healing are manifold (Fig. 4). First, it needs to be re-emphasized that chronic wounds constitute a highly heterogeneous group of diseased tissue. Depending on the primary etiological agent, there are different wound types, such as venous leg ulcers, pressure ulcers, and diabetic foot ulcers. Wound size or volume, respectively, is an essential factor to consider in terms of practical gas plasma exposure. Wound location is critical, e.g., a wound in a non-flat body location hinders the application of flat DBD (Fig. 6, bottom image) devices that will not deliver the plasma appropriately. Wounds also differ in age and chronification, already affecting traditional wound care [192,193]. Additional co-morbidities and the immune status are of therapeutic relevance as well [194]. Wound colonization profiles and antimicrobial resistance (AMR) against drugs are known to be parameters impeding proper wound healing [195]. Additionally, the standard of care in wound treatment differs substantially between and even within countries. These parameters make it difficult to gather large cohorts of patients with similar wound traits. Against this characterization matrix, a similar degree of variation can be outlined for gas plasma therapy. This includes the duration, i.e., the length of treatment, usually ranging from seconds to minutes per square centimeter. While companies make recommendations, based on relatively little evidence, there is no consensus on the frequency of treatment, i.e., how often per week and which treatment breaks should plasma be applied. It is also unknown whether some areas within the wound (e.g., the margins or heavily infected areas) should be preferentially exposed to gas plasma. From a technical perspective, there are also no clear recommendations on which device performs best for which type of wound. Given the promising pre-clinical data on optimizing healing responses via changing the ROS/RNS composition through feed gas modulation in plasma jets (Fig. 6, upper image) as described above for many animal models, there is a good chance of further improving wound healing in the clinics in the future with optimized gas settings. It is also not explored whether there is an optimal timing of plasma therapy within the existing standard of care regimens. Most importantly, identifying determinants of when to start and stop plasma treatment needs to be identified.

**Fig. 5:**
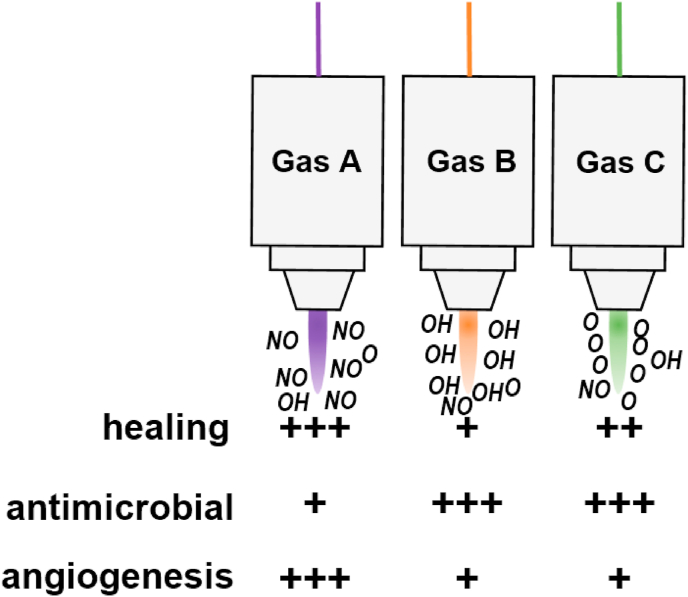
Hypothetical matrix showing the effects of three different feed gas supplements into a plasma jet. Pre-clinical results suggest some gas plasma-derived ROS/RNS mixtures to be more beneficial over others in terms of specific biological outcomes, making future clinical studies on such modifications a promising endeavor. Gas plasma conditions are shown to be enriched for NO (nitric oxide), OH (hydroxyl radicals), and atomic oxygen (O) for schematic purposes; gas plasmas are always mixtures of ROS/RNS but can be focused towards a relative increase in production of specific types of ROS/RNS.

**Fig. 6:**
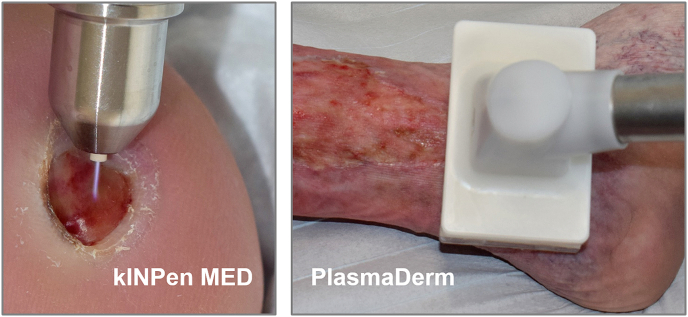
Photographs of the clinical application of the *kINPen MED* and *PlasmaDerm* device in supportive wound care.

Several testable hypotheses can be directly deduced from this gas plasma-wound matrix, which needs the enthusiasm of medical practitioners within large wound centers to be addressed, for instance: Are aged wounds in need of different gas plasma treatment frequencies? Do some microorganisms that impede healing to an overarching extent succumb more readily to specific gas plasma-derived ROS/RNS mixtures within wounds? For best results, does the wound size linearly correlate to treatment time, or should some wound regions receive preferential exposure over others? Given the game-changing clinical results gas plasma therapy had provided so far, such questions are not only an academic endeavor but have practical consequences for many patients worldwide, especially those failing to respond to standard wound therapies.

## 7 Future concepts in gas plasma-stimulated wound healing

The clinical application of gas plasma wound therapy is promising. Future concepts in this field are related to technical, scientific, and clinical horizons. Compared to other physical modalities in medicine, such as a century of experience in radiotherapy, clinical gas plasma technology is still in its infancy. To make devices and treatment intensities comparable in the long run, the definition of a unit system – comparable to a “dose” - would be helpful. At the moment, the multimodal nature of gas plasmas, the differences in device geometries, and the lack of identified key mediators hamper such efforts. For plasma jets, multijet systems are needed that are ideally scalable between smaller and larger areas at equivalent energy depositions to facilitate the treatment of extensive wound regions in a reasonable amount of time to remain cost-effective. Given the high incidence of chronic wounds, this is an essential factor to consider. Ongoing work explores hand-held, battery-operated gas plasma devices. Such approaches may revolutionize wound care of immobile patients during home care. Furthermore, combining plasma devices with specialized units for continuous and real-time monitoring of both plasma characteristics and the target during the treatment will lead to a new generation of gas plasma-based therapeutic systems [196].

Scientifically, gas plasma technology is attractive to redox biology and medicine because of its true multi-ROS/RNS nature. In the gas phase, these species can be characterized very well [45]. While disentangling individual species types in tissues is limited with current techniques available, many processes, especially inflammation, are known to involve several redox chemistry pathways [60], making gas plasmas an attractive option to mimic inflammatory redox environments. Considering the hormetic effect of ROS/RNS, it would be beneficial to perform more parameter studies *in vivo* on treatment duration and frequency to identify the ROS/RNS doses and molecular markers that determine the boundary between oxidative eustress (promoting healing) and distress (hampering healing). Such models would be highly tutorial to redox research in general and wound research and inflammation research specifically. Knockout mice lacking, e.g., macrophages or Nrf2 would be of great interest in this regard. Further unaddressed questions are the diffusion trajectories of species in tissues. For instance, gas plasma exposure promotes nitrotyrosine formation [197], which could be tested in the skin and wound tissues in a dose (treatment time) dependent manner. Other relevant questions relate to NET formation, cytokine and chemokine release, and senescent cell apoptosis in gas plasma-treated experimental wounds investigated soon after exposure. These experiments would facilitate the understanding of how the redox system can be exploited therapeutically.

Clinically, there is a great need for more parameter studies to link gas plasma devices, exposure durations and frequencies, infection status, wound location, and treated sites within to wound to objective responses. International guidelines should be proposed and continuously developed to outline effective clinical gas plasma therapy. Notably, more than one hundred *kINPen MED* devices are already used daily in clinical centers across central Europe for patient care, but structured studies and reports on such clinical experience are scarce. These, however, are utterly needed to drive standardization procedures. Also, head-to-head trials of marketed devices are inevitably needed to continue developing topical ROS/RNS gas plasma therapies optimized towards specific wound applications. Especially for jet plasma systems that can be operated using different feed gas compositions generating distinct ROS/RNS mixtures, pre-clinical evidence is promising [81,90,91,93,98] to hypothesize wound condition-optimized gas plasma conditions yet to be identified (Fig. 5). Moreover, wound material samples hold excellent promises to expand the understanding of redox control in the inflammatory wound environment. Chemokine and cytokine quantification of selected targets is affordable today, and pre-selected samples could be characterized by mass spectrometry for identifying redox-regulated protein signatures in wounds. Standardized sampling protocols and databases should be published to support such a process. Combination therapies are another promising approach to improve gas plasma-aided wound healing. Experienced centers combine this therapy with vacuum therapy for many years now already with success, but published evidence on combination treatments is not available so far. Referring back to the initially described concept of redox regulation in wound healing proposed decades ago [8,10,11], it might be intriguing to combine gas plasma with oxygen for wound therapy [198].

## 8 Conclusion

Gas plasma systems are long known to have profound antimicrobial activity, suggesting their suitability to combat defective wound healing in a symptomatic and not causal therapy. Today, compelling preclinical and evolving clinical evidence underlines the favorable healing responses of gas plasma-stimulated wounds. Nevertheless, concrete clinical recommendations can hardly be made today due to the different systems and applications used and the data situation. Overall, the evidence-based findings are hopeful but still insufficient to extract guideline-directed gas plasma therapies across all types of wounds and etiologies. However, from a scientific point of view, it is notable that several recent reports indicated wound healing-promoting effects of the gas plasma-derived ROS/RNS independent of antiseptic plasma effects. While the ROS/RNS mixtures of gas plasma are complex and hence challenging for distilling single modes of action as of now, these encouraging findings may nevertheless form the foundation of the exciting paradigm of redox regulation through gaseous ROS/RNS in medicine.

## Funding statement

This work was funded by the German 10.13039/501100002347Federal Ministry of Education and Research (10.13039/501100002347BMBF), grant numbers 03Z22DN11 and 16GW0345 (PlasFect). The sponsor had no role in writing the article or in the decision to submit the article for publication.

## Declaration of competing interest

The majority of research that served as a scientific basis for development and CE certification of the *kINPen MED* as a medical device sold by the neoplas MED GmbH (Greifswald, Germany) has been realized by work at the Leibniz Institute for Plasma Science and Technology (INP) before commercialization. None of the authors of this article has a direct or financial relationship with this company. Steffen Emmert received speaker honoraria from Cinogy GmbH (Duderstadt, Germany).
